# Preemptive rapid antigen test as an emergency measure during coronavirus disease 2019 outbreaks

**DOI:** 10.3389/fpubh.2026.1764471

**Published:** 2026-02-23

**Authors:** Yaping Pan, Yong Liu, Ye Jiang, Jin Cai

**Affiliations:** Naval Medical Center, Naval Medical University, Shanghai, China

**Keywords:** acute respiratory infectious diseases, coronavirus disease 2019, emergency measures, outbreaks, rapid antigen test, reverse transcriptase polymerase chain reaction

## Abstract

**Background:**

We acknowledge that the rapid antigen test (RAT) has several advantages, including faster results, cost-effectiveness, and suitability for on-site self-testing. However, there is still controversy regarding the performance of the RAT for coronavirus disease 2019 (COVID-19). The aim of this study was to evaluate RAT screening in a COVID-19 outbreak situation.

**Methods:**

In this study, we developed a preemptive testing model in which a RAT was immediately followed by reverse transcriptase polymerase chain reaction (RT-PCR) during a single screening in a high-density community outbreak to rapidly prevent transmission. The RAT and RT-PCR were performed using the Flowflex^™^ SARS-CoV-2 Antigen Test Kit and the SARS-CoV-2 Nucleic Acid Test Kit, respectively. Furthermore, we retrospectively investigated diagnostic data from a total of 813 participants. Then, we analyzed sensitivity and specificity using RT-PCR as the reference method. In addition, we compared our data with those from another published study involving serial screening using the chi-squared test, in which 541 samples were analyzed.

**Results:**

Our study showed that the sensitivity and specificity of the RAT were 0.82 and 1.00, respectively. We found a slightly higher false-negative rate, corresponding to decreased sensitivity, in our data compared to the previous study; however, the difference was not statistically significant. In addition, there was no statistically significant difference in the false-positive rate, corresponding to specificity, between the two studies.

**Conclusion:**

The current results indicate that a RAT with a single screening does not pose an additional risk of inaccurate diagnosis of COVID-19 compared to serial screening. The strategy of a preemptive RAT might be an effective emergency measure to prevent COVID-19 transmission at an early stage of an outbreak.

## 1 Introduction

Recently, coronavirus disease 2019 (COVID-19), caused by severe acute respiratory syndrome coronavirus 2 (SARS-CoV-2), has entered an endemic stage with seasonal distribution globally (1). However, transmission can still occur extremely fast in high-density communities (2). It continues to have a great impact on personal health, particularly among pregnant women, young children, older adults, and populations with chronic underlying diseases (3, 4). There are some barriers to the rapid and accurate detection of SARS-CoV-2 due to its intrinsic and constant mutation (5). Consequently, conflicting results may arise, leading to confusion in subsequent prevention and treatment strategies.

A positive detection of SARS-CoV-2 nucleic acid by reverse transcriptase polymerase chain reaction (RT-PCR) is the primary standard for the diagnosis of COVID-19 (6). This method requires both professional laboratories and trained personnel, so it may not be widely used outside of large hospitals or medical institutions (7, 8). In addition, the detection process is not well suited for high-throughput screening within a short period of time (8). Although somewhat less sensitive and specific than RT-PCR, the rapid antigen test (RAT) for the diagnosis of COVID-19 offers several advantages, including faster results, cost-effectiveness, and suitability for on-site self-testing, providing a trade-off between clinical performance, speed, and accessibility (8, 9). The RAT enables the earlier implementation of emergency measures, including isolation precautions, contact tracing, and antiviral treatment, which can help prevent transmission more rapidly (10, 11). Therefore, the RAT has been recommended as a triage tool before RT-PCR testing (12) and may be preferable in emerging circumstances, such as for close contact, isolated individuals, or symptomatic cases during a COVID-19 outbreak.

Despite these advantages, there is limited clear evidence that the RAT can serve as an emergency measure during COVID-19 outbreaks, especially for large-scale screening in high-density communities. A previously published study evaluated the performance of the RAT in the context of hospital COVID-19 outbreaks, conducting serial dual screening with both RT-PCR and the RAT across different hospital units (13). However, the diagnostic performance of the RAT during a single screening in a single outbreak has not yet been reported.

The aim of this study was to evaluate the performance of a preemptive RAT during a single screening in a high-density community outbreak. We retrospectively analyzed our data in comparison with the previous study described above. Moreover, we discussed the aspect serving as an applicable emergency measure in COVID-19 outbreak situation.

## 2 Methods

### 2.1 Study design and population

This retrospective study was carried out among close contacts during a COVID-19 outbreak in a high-density community in Hebei province, China. Individuals who agreed to participate underwent a preemptive RAT immediately followed by RT-PCR between 1 and 4 December 2022 to rapidly prevent transmission. Data were retrospectively collected from medical service records, and a total of 813 participants aged 18 years and older were included. However, cases for which results of either the RAT or RT-PCR were unavailable were excluded. This retrospective study was approved by the Medical Ethics Committee of the Naval Medical Center of PLA, China, and was exempted from the requirement for informed consent, as the data contained no identifying information of participants (reference number: AF-HEC-074; grant date: 15/07/2025).

### 2.2 Study procedure

Approximately 72 h after the first positive case of SARS-CoV-2 was identified in the community through periodic RT-PCR screening on 1 December 2022, we performed duplicate RATs on oropharyngeal swabs, immediately followed by RT-PCR, on 4 December 2022. Individuals who tested positive by preemptive RATs were promptly managed with emergency measures, including isolation precautions, contact tracing, and antiviral therapy, while those who tested negative remained as close contacts under personal protective measures. Once cases were confirmed by RT-PCR, these measures were immediately implemented. For the emergency screening, true-positive participants and false-negative cases were considered positive for management purposes, while true-negative and false-positive individuals were treated as close contacts, in accordance with the Protocol for Prevention and Control of COVID-19 in China (Edition 9) (14).

### 2.3 Sample collection and handling

Samples were placed in 3 mL of viral transport medium and transported at 2–8 °C for processing within a few hours. All specimens were processed in biosafety level-3 and enhanced biosafety level-2 facilities using full personal protective equipment.

### 2.4 Laboratory analysis

We used the Flowflex^™^ SARS-CoV-2 Antigen Test Kit (Hangzhou ACON Biotech Co. Ltd., Zhejiang, China) for the RAT of SARS-CoV-2. The kit is a lateral flow chromatographic immunoassay for the qualitative detection of the nucleocapsid protein antigen in respiratory specimens. RATs were performed according to the manufacturer’s protocol. A test was considered positive when two colored lines—control (C) and test (T)—appeared within 15 min.

Viral RNA extraction and real-time RT-PCR for SARS-CoV-2 were conducted using the SARS-CoV-2 Nucleic Acid Test Kit (Wuhan EasyDiagnosis Biomedicine Co., Ltd., Hubei, China), which targets the ORF1ab and N genes. Only samples with a cycle threshold (Ct) value less than 40 for both target genes were considered positive for RT-PCR. The results of the RAT were not available to those performing the RT-PCR, and vice versa.

### 2.5 Statistical analysis

We used SPSS 25.0 to calculate the incidence rate (based on the reference method, as described below), sensitivity, specificity, positive predictive value (PPV), negative predictive value (NPV), and accuracy of the RAT, using RT-PCR as the reference method. Moreover, we compared our data with a previously published study in which the RAT was performed on suspected patients during hospital COVID-19 outbreaks from October 2020 to January 2021 (13). We analyzed the relative risk (RR) of false-negative and false-positive RAT results between our real-world study and the previous study using the chi-squared test to evaluate sensitivity and specificity, respectively. We reported confidence intervals (CIs) for each statistical description if necessary, and differences were considered statistically significant at a *p*-value of < 0.05.

## 3 Results

### 3.1 Performance of the RAT in our real-world study during a COVID-19 outbreak

In total, 123 of 813 participants in the real-world study tested positive by both the RAT and RT-PCR, yielding 662 consistent negative results. In addition, 27 participants had conflicting results, testing negative by the RAT but positive by RT-PCR, and one participant tested positive by the RAT but negative by RT-PCR. No discrepancies were observed between duplicate RATs during the screening in this study. Using RT-PCR as the reference method, the incidence rate, sensitivity, specificity, PPV, NPV, and accuracy of the RAT as a diagnostic test were 0.18 (95% CI: 0.16–0.21), 0.82 (95% CI: 0.76–0.88), 1.00 (95% CI: 0.99–1.00), 0.99 (95% CI: 0.98–1.00), 0.96 (95% CI: 0.95–0.98) and 0.97 (95% CI: 0.95–0.98), respectively (Table 1).

**Table 1:** Performance of the RAT in COVID-19 outbreak situations using RT-PCR as the reference method.

Authors	Sample size	True positive	False positive	False negative	True negative	Incidence rate (95% CI)	Sensitivity (95% CI)	Specificity (95% CI)	PPV (95% CI)	NPV (95% CI)	Accuracy (95% CI)
Pan et al.	813	123	1	27	662	0.18 (0.16–0.21)	0.82 (0.76–0.88)	1.00 (1.00–1.00)	0.99 (0.98–1.00)	0.96 (0.95–0.98)	0.97 (0.95–0.98)
Aranaz-Andrés et al. (13)	541	25	1	5	510	0.055 (0.036–0.075)	0.83 (0.69–0.98)	1.00 (0.99–1.00)	0.96 (0.88–1.00)	0.99 (0.98–1.00)	0.99 (0.98–1.00)

RAT, rapid antigen test; COVID-19, coronavirus disease 2019; RT-PCR, reverse transcriptase polymerase chain reaction; PPV, positive predictive value; NPV, negative predictive value; CI, confidence interval.

In the previous study, a total of 541 samples were analyzed by RT-PCR followed by the RAT, yielding 25 consistent positive results and 510 consistent negative results. In addition, six samples showed conflicting results: Five tested negative by the RAT but positive by RT-PCR, and one tested positive by the RAT but negative by RT-PCR (13). According to the results of the previous study, the incidence rate, sensitivity, specificity, PPV, NPV, and accuracy of the RAT as a diagnostic test are presented in Table 1.

### 3.2 Comparison of RAT performance between our real-world study and a previously published study during a COVID-19 outbreak

We found that the false-negative rate, corresponding to sensitivity, of the RAT in our model was slightly higher compared to the previous study, but the difference was not statistically significant (0.18 [95% CI: 0.12–0.24] vs. 0.17 [95% CI: 0.03–0.31], RR: 1.08 [95% CI: 0.45–2.58], *p* = 0.862). In addition, the false-positive rate, corresponding to specificity, of the RAT showed no statistically significant difference between the two studies (0.002 [95% CI: 0.000–0.004] vs. 0.002 [95% CI: 0.000–0.006], RR: 0.77 [95% CI: 0.05–12.29], *p* = 0.853), as shown in Table 2.

**Table 2:** Comparison of RAT performance between our real-world data and a previous study in COVID-19 outbreak situations.

RAT performance	Pan et al.	Aranaz-Andrés et al. (13)	RR (95% CI)	*P*-value
False-negative rate	0.18 (0.12–0.24)	0.17 (0.025–0.31)	1.08 (0.45–2.58)	0.862
False-positive rate	0.0015 (0–0.0045)	0.0020 (0–0.0058)	0.77 (0.05–12.29)	0.853

RAT, rapid antigen test; RR, relative risk; CI, confidence interval.

### 3.3 Distribution of Ct values in RT-PCR samples with discordant RAT results

Of the 27 cases with negative RAT and positive RT-PCR results for ORF1ab, 13 had Ct values between 30 and 35, reinforcing their classification as false negatives. Overall, almost half of the false-negative cases had Ct values between 30 and 35, with an average of 33.76, in this study, as shown in Table 3. In addition, the Ct value of the single case with a positive RAT but negative RT-PCR was not recoded, although it was reported to be above 40.

**Table 3:** Distribution of Ct values for ORF1ab in positive RT-PCR samples with discordant negative RAT results.

Range of Ct values	Ct values of ORF1ab	Sample size
< 15	/	0
15–20	18.75	1
20–25	23.24 ± 1.61	3
25–30	27.81 ± 1.59	7
30–35	33.76 ± 0.90	13
35–40	36.57 ± 1.13	3

Ct, cycle threshold; COVID-19, coronavirus disease 2019; RAT, rapid antigen test; RT-PCR, reverse transcriptase polymerase chain reaction.

## 4 Discussion

Emergency measures in a COVID-19 outbreak, including rapid detection of SARS-CoV-2, early isolation of positive individuals, and effective therapy of confirmed cases, are necessary to prevent the rapid spread of the virus. Although RATs have been recommended for quick detection, there is still controversy regarding their performance, as RT-PCR is the primary diagnostic method (15–18). In our model, a preemptive RAT strategy involving a single screening for emergency situations during a COVID-19 outbreak was developed. We analyzed our real-world data and compared them with a previous study that used serial screening in hospital COVID-19 outbreaks, using the chi-squared test. The growing body of evidence is supported by the comparison of our real-world data with that of the previous study.

Our results demonstrated that the RAT with a single screening for the diagnosis of COVID-19 during outbreak situations, when referenced against RT-PCR, yielded a substantially acceptable false-negative rate compared to the previous study with multiple screenings. Although the difference was not statistically significant, the slightly higher false-negative rate in our study indicated a somewhat lower sensitivity, which might be attributed to the lower screening frequency compared to the previous study. However, the sensitivity of the RAT for the diagnosis of COVID-19 in this study was comparable to that reported in other published studies (19–23), ranging from 0.61 (95% CI: 0.52–0.69) (23) to 0.98 (95% CI: 0.91–1.00) (19). In addition, our study showed that almost half of the false negatives had Ct values between 30 and 35, compared to 80% (4 of 5) with Ct values between 25 and 30 in the previous study (13). A possible reason for this is the difference in detection thresholds: Ct values below 40 and 35 were considered positive for RT-PCR in the current and previous studies, respectively (13). In addition, it has been reported that the sensitivity of the RAT as a diagnostic test appears to be positively associated with the incidence rate of COVID-19 in the study population, as previously described (24). Certainly, additional correlation analysis between the two factors is needed. Notably, previous studies have reported that sensitivity is positively related to the amount of viral RNA in specimens, as indicated by PCR Ct values (13, 25, 26). Therefore, proper swab sampling to reach the designated site and efforts to lower the detection limit are necessary to improve test performance.

Notably, the RAT exhibited excellent specificity, reaching up to 99%, with an extremely low false-positive rate, nearly equivalent to that observed in the previous study using serial screening. Moreover, the specificity of the RAT for the diagnosis of COVID-19 in this study was consistent with other published studies (19–23), ranging from 0.99 (95% CI: 0.97–1.00) (19) to 1.00 (95% CI: 1.00–1.00) (23). This high specificity offers the advantage that a positive RAT result could be interpreted as indicative of COVID-19 infection promptly. Based on these findings, the RAT could serve as a preferred method for mass screening in high-density populations during a COVID-19 outbreak, rather than serving solely as a complementary tool to RT-PCR. This approach requires less time to implement emergency measures, including isolation precautions, contact tracing, and antiviral treatments. Consequently, we could prevent transmission at a rate that keeps pace with the spread of SARS-CoV-2. We presumed that developing a preferred RAT for emergency responses to another acute respiratory infectious disease would be feasible.

From a public health perspective, a preemptive RAT contributes to strengthening prevention and control measures at an earlier stage, including mask-wearing, physical distancing, and hygiene management. It also provides evidence that personnel classification and then shunt could be performed shortly, slowing down the spread of COVID-19 as well as acute respiratory infectious diseases. However, pooling samples for a large-scale screening by RT-PCR brings pending positive results of pooled samples that need more time to obtain individual outcomes (27). Therefore, RT-PCR for pooled samples would be applied to populations with relatively limited cases and a lower risk of transmission, or in resource-constrained circumstances (28). For comparison, the RAT could overcome these disadvantages, and may play an even more important role in mass screening as part of emergency measures during outbreak situations.

This study has a few limitations. First, in comparison with the previously published study, there might be some differences in the epidemic population, outbreak stage, and study design. Second, more attention should be given to other factors that could affect RAT accuracy, such as detection reagents, practitioner skills, and the inspection process (29). Third, as this observational study involved a limited population, the findings need to be confirmed with a large number of cases in future intervention trials.

## 5 Conclusion

Overall, our results indicate that a RAT with a single screening does not pose an additional risk of inaccurate COVID-19 diagnosis in outbreak situations compared to serial screening. A preemptive RAT might be an effective emergency measure to prevent COVID-19 transmission at an earlier stage. We provide new evidence that the strategy of a preemptive RAT could be applied for emergency responses to outbreaks of acute respiratory infectious diseases. We also look forward to further high-quality research to confirm and expand upon our findings.

## Data Availability

The original contributions presented in the study are included in the article/supplementary material, further inquiries can be directed to the corresponding author.
